# Supplementary material for: Dynamic transcriptomic profiles of zebrafish gills in response to zinc supplementation
Source: BMC Genomics. 2010 Oct 11;11:553. doi: 10.1186/1471-2164-11-553 (PMC3091702; doi:10.1186/1471-2164-11-553)
Supplement: Additional file 2 — Interactive Direct Interaction Network representing the molecular interactions between zinc, copper, iron, calcium and proteins encoded by transcripts changed by zinc supplementation. Mini web-site containing index.html and hyperlinked pages in subdirectory describing a Direct Interaction Network automatically generated based on curated interactions contained within the proprietary PathwayArchitect database. Ovals represent proteins and the circles symbolize metal ions. Objects are coloured by their abundance in zebrafish at the time-point they were significantly different from the control is a scale from -4 fold (dark green) to +4 fold (dark red). Where significant differences were found at more than one time-point, the colour overlay shows expression at the first instance. Dark blue squares denote 'binding', and light blue squares 'expression'; green squares stand for 'regulation', green diamonds for 'metabolism', and green circles for 'promoter binding'. Arrow heads indicate directionality of the interaction where annotated. All nodes and edges can be further interrogated by selecting the relative area of the image. [file 1471-2164-11-553-S2.zip › PathwayArchitect Zn xs DIN/73087.html]

# PROTEIN: PRSS2

|  |  |
| --- | --- |
| Name | PRSS2 |
| Type | PROTEIN |
| Description | protease, serine, 2 (trypsin 2) |
| Note | This gene encodes a trypsinogen, which is a member of the trypsin family of serine proteases. This enzyme is secreted by the pancreas and cleaved to its active form in the small intestine. It is active on peptide linkages involving the carboxyl group of lysine or arginine. This gene and several other trypsinogen genes are localized to the T cell receptor beta locus on chromosome 7. |
| Alias | TRY8 |
|  | Ta |
|  | Pretrypsinogen II |
|  | Tesp4 |
|  | anionic trypsinogen |
|  | TRYP |
|  | trypsin II |
|  | Prss2 |
|  | Try2 |
|  | TRY2 |
|  | pancreatic trypsin 2 |
|  | PRSS2 |
|  | trypsin 2 |
|  | Ptryss2 |
|  | Anionic trypsinogen |
|  | protease, serine, 2 |
|  | pancreatic trypsin II |
|  | TRYP2 |
|  | trypsinogen 2 |


---

|  |  |
| --- | --- |
| GO Component | extracellular region |


---

|  |  |
| --- | --- |
| GO ID | GO:0004252 |
|  | GO:0008233 |
|  | GO:0007586 |
|  | GO:0004263 |
|  | GO:0046872 |
|  | GO:0006508 |
|  | GO:0016787 |
|  | GO:0005509 |
|  | GO:0005576 |
|  | GO:0004295 |


---

|  |  |
| --- | --- |
| MIM | MIM:601564 |


---

|  |  |
| --- | --- |
| Connectivity | 95 |


---

|  |  |
| --- | --- |
| Entrez ID | 22072 |
|  | 5645 |
|  | 25052 |


---

|  |  |
| --- | --- |
| Agilent ID | A\_44\_P554904 |
|  | A\_53\_P156110 |
|  | A\_23\_P335838 |
|  | A\_32\_P94444 |
|  | A\_23\_P310274 |
|  | A\_51\_P418901 |
|  | A\_53\_P145732 |


---

|  |  |
| --- | --- |
| Cellular Localization | Extracellular region |


---

|  |  |
| --- | --- |
| Pathway | Zn xs inventory |
|  | Zn xs DIN |


---

|  |  |
| --- | --- |
| GO Process | proteolysis and peptidolysis |
|  | proteolysis |
|  | digestion |


---

|  |  |
| --- | --- |
| UniGene | Mm.276926 |
|  | Hs.367767 |
|  | Rn.1584 |


---

|  |  |
| --- | --- |
| Affymetrix Probeset ID | 1370126\_at |
|  | 1417682\_a\_at |
|  | 1433459\_x\_at |
|  | 1433573\_x\_at |
|  | 1435507\_x\_at |
|  | 205402\_x\_at |
|  | 216470\_x\_at |
|  | 92873\_f\_at |
|  | g4506144\_3p\_x\_at |
|  | g4506146\_3p\_x\_at |
|  | L00131cds\_s\_at |
|  | U66061\_cds3\_at |
|  | V01274\_at |
|  | x04574\_s\_at |
|  | 101340\_at |
|  | 32796\_f\_at |
|  | M27602\_f\_at |
|  | 1380183\_at |


---

|  |  |
| --- | --- |
| EC Number | EC 3.4.21.4 |


---

|  |  |
| --- | --- |
| GO Function | hydrolase activity |
|  | peptidase activity |
|  | trypsin activity |
|  | chymotrypsin activity |
|  | serine-type endopeptidase activity |
|  | metal ion binding |
|  | calcium ion binding |


---

|  |  |
| --- | --- |
| Nucleotide | NG\_001333 |
|  | AK003082 |
|  | L00130 |
|  | BC005814 |
|  | K03042 |
|  | NM\_012729 |
|  | AB017030 |
|  | BC030260 |
|  | V01274 |
|  | L00131 |
|  | NM\_002770 |
|  | K03044 |
|  | X04577 |
|  | NM\_009430 |
|  | AF009664 |
|  | M27602 |
|  | AY052784 |
|  | X04574 |
|  | U66061 |
|  | CH236959 |
|  | K03043 |
|  | AE000665 |


---

|  |  |
| --- | --- |
| Protein | CAA24581 |
|  | AAH05814 |
|  | NP\_033456 |
|  | AAC13351 |
|  | CAA28245 |
|  | NP\_036861 |
|  | CAA28243 |
|  | AAL14244 |
|  | AAA61232 |
|  | P07146 |
|  | BAB22554 |
|  | AAB69089 |
|  | AAH30260 |
|  | P07478 |
|  | AAA98517 |
|  | AAC80209 |
|  | P00763 |
|  | EAL23774 |
|  | NP\_002761 |
|  | BAA74759 |


---

|  |  |
| --- | --- |
| Organism | Mammal |


---

|  |  |
| --- | --- |
| Location | chromosome 6, 6 B1 (Mus musculus) |
|  | chromosome 4, 4q22 (Rattus norvegicus) |
|  | chromosome 7, 7q34 (Homo sapiens) |


---

|  |  |
| --- | --- |
